# Advances in Lymphoma Molecular Diagnostics

**DOI:** 10.3390/diagnostics11122174

**Published:** 2021-11-23

**Authors:** Igor Age Kos, Lorenz Thurner, Joerg Thomas Bittenbring, Konstantinos Christofyllakis, Dominic Kaddu-Mulindwa

**Affiliations:** Department of Hematology, Oncology, Clinical Immunology, Rheumatology, Medical School, University of Saarland, 66424 Homburg, Germany; igor.kos@uks.eu (I.A.K.); lorenz.thurner@uks.eu (L.T.); joerg.thomas.bittenbring@uks.eu (J.T.B.); konstantinos.christofyllakis@uks.eu (K.C.)

**Keywords:** lymphoma diagnostics, molecular diagnostics, non-Hodgkin lymphoma, sequencing

## Abstract

Lymphomas encompass a diverse group of malignant lymphoid neoplasms. Over recent years much scientific effort has been undertaken to identify and understand molecular changes in lymphomas, resulting in a wide range of genetic alterations that have been reported across all types of lymphomas. As many of these changes are now incorporated into the World Health Organization’s defined criteria for the diagnostic evaluation of patients with lymphoid neoplasms, their accurate identification is crucial. Even if many alterations are not routinely evaluated in daily clinical practice, they may still have implications in risk stratification, treatment, prognosis or disease monitoring. Moreover, some alterations can be used for targeted treatment. Therefore, these advances in lymphoma molecular diagnostics in some cases have led to changes in treatment algorithms. Here, we give an overview of and discuss advances in molecular techniques in current clinical practice, as well as highlight some of them in a clinical context.

## 1. Introduction

Lymphomas are a broad group of neoplasms involving the lymphoid tissues, divided into two large subgroups: Hodgkin lymphoma (HL) and non-Hodgkin lymphoma (NHL) [1]. Whereas HL is histologically defined by the presence of malignant Hodgkin/Reed–Sternberg cells surrounded by a diversity of non-malignant and inflammatory cells, NHLs comprise a wide class of lymphoid neoplasms that evolve from the clonal expansion of mature B, T and natural killer (NK) cells in different stages of development [2,3]. Depending on the lineage from which they evolve, NHLs are further classified into B, T and NK lymphomas [1].

NHLs are the most prevalent hematopoietic neoplasms, accounting for approximately 4.3% of all cancer diagnoses [4,5]. Of them, B cell NHL accounts for approximately 30% of all lymphoid neoplasms, followed by HL (8%) and T/NK neoplasms (5%) [6]. Diffuse large B cell lymphoma (DLBCL) is the most common subtype of NHL and accounts for approximately 25% of all NHL cases.

NHLs can arise from mutations in almost every phase of lymphocyte differentiation; thus, many subtypes of lymphomas share common characteristics with their non-malignant equivalents (cell of origin). These similarities in morphology and immunophenotype are very important for the diagnosis and classification of lymphomas [7]. However, due to differences in clinical presentation, NHLs are often further classified into aggressive and indolent [8]. Aggressive B cell lymphomas include mainly DLBCL, follicular lymphoma (FL), grade III, and Burkitt lymphoma (BL), whereas indolent lymphomas embrace many other entities, such as small lymphocytic lymphoma (SLL)/chronic lymphocytic leukemia (CLL), lymphoplasmocytic lymphoma (LPL), multiple myeloma (MM), hairy cell leukemia (HCL), follicular lymphoma (grade I and II) and marginal zone lymphoma (MZL) [8]. Of note, some entities, such as mantle cell lymphoma (MCL), may present either with an aggressive or indolent clinical course [9].

B and T cells harbor unique signatures regarding their receptors and their respective genes [10]. B cell receptors are extremely variable proteins, that due to individual-cell genetic recombination in variable (V), diversity (D) and joining (J) segments have the possibility to recognize an enormous number of antigens [11]. In addition, B cells undergo two late changes to their deoxyribonucleic acid (DNA) during the germinal center reaction: hypermutation and class switch. This heterogeneity is extremely important for the diversity of the immune system; however, it might trigger alterations which may lead to lymphomagenesis [7].

A broad number of genetic alterations, such as chromosomal translocations and alterations, somatic mutations and epigenetic alterations, are seen in virtually all subtypes of lymphomas. The characterization of these changes is of clinical importance, as it could enable targeted therapy [12].

Even though the clinical features, morphology and immunophenotype of lymphomas are mainstays of the diagnosis and classification of lymphomas, the identification of their genetic alteration has gained more attention in recent years [13]. The World Health Organization (WHO)’s classification of lymphoid neoplasms has gone through periodical revisions in order to encompass clinical and molecular advances, including expanding the number of key genetic changes observed in this group of diseases [1].

The purpose of this review is to provide an overview of these alterations and advances in lymphoma molecular diagnostics with high relevance in clinical practice.

## 2. Molecular Diagnostics and Genetic Alterations with Clinical Relevance

### 2.1. Common Genetic Alterations with Diagnostic Significance

The identification of recurrent molecular alterations in NHLs has been performed over many years and gained specific importance for diagnostic purposes. An often-cited example is the identification of aberrations involving the immunoglobulin (Ig) genes [14]. Since the IgH gene is located on the chromosome 14q32, which is an especially active genetic area of B lymphocytes, changes involving this chromosome are often related to lymphomagenesis [15]. Rearrangements of *MYC*, a gene located on chromosome 8 which encodes a potent proto-oncogene protein, are an important genetic characteristic of BL. They can be identified in virtually all cases of BL [16]. This rearrangement is caused by a translocation involving chromosome 14, t(8;14)(q24:q32), in 80% of cases [17]. It is not entirely coincidental that the other 20% of translocations involve Ig light chain genes, more specifically between chromosomes 2 and 8, t(2;8)(p12;q24), or chromosomes 8 and 22, t(8;22)(q24;q11). Other translocations involving chromosome 14 are often used for diagnostic purposes. In FL, translocation t(14;18) (q32; q21) approximates the active enhancer of the IgH to the BCL2 gene, causing the augmented expression of the BCL2 protein, resulting in the inhibition of apoptosis and excessive proliferation [18]. Even though this alteration is not specific to FL, it is found in 80–96% in patients with FL depending on the method, thus still having diagnostic importance [19,20]. The overexpression of the cyclin D1 protein, a regulator of the early phases of the cell cycle through the inactivation of the tumor suppressor gene retinoblastoma, plays an important role in the pathogenesis of MCL [21]. The translocation t(11;14)(q13;q32) juxtaposes the IgH gene enhancer on 14q32 to the cyclin D1 gene on 11q13, leading to overexpression. This translocation can be found, depending on the method, in almost all cases of MCL, helping physicians in clinical practice to confirm the diagnosis [22].

Apart from chromosomal changes, other genetic aberrations such as point mutations or small insertions also play an important role in lymphomagenesis, and are important tools for the diagnosis of B cell lymphomas. Advances in molecular methods have led to an expansion of the current knowledge regarding recurrent genetic alterations in this group of diseases [23]. Consequently, over the last few years, the WHO classification has included and highlighted some new important genetic alterations, which can be especially helpful in cases of diagnostic uncertainty and even more so in the setting of clinical trials, where patient selection is fundamental [1]. Some examples of these alterations are the detection of *BRAF* V600 E/K in HCL [24] and *MYD88* p.L26P mutations in lymphoplasmacytic lymphoma and Waldenstrom’s macroglobulinemia [25], which are considered to be disease-defining alterations. Both of these changes were incorporated into clinical practice due to the emergence of new techniques, in this case next-generation sequencing (NGS) studies [1].

Interestingly, the association of some of these diseases with their respective genetic alterations is so strong that the absence of the expected genetic constellation in the presence of compatible phenotypical presentation may lead to diagnostic challenges. In these cases, further investigation of the mutational status may bring additional benefit.

One example is Burkitt-like lymphoma with 11q aberrations, a disease that resembles BL but lacks the *MYC* rearrangement. It was recently added as a provisional entity in the WHO 2016 classification of lymphoid neoplasms [1,26]. Similarly, a hairy cell leukemia variant that presents with *MAPK1* mutations in 50% of the cases was also added [27], as was predominantly diffuse FL with 1p36 deletion, which often presents as a localized inguinal mass and lacks *BCL2* rearrangement [28]. Table 1 summarizes some genetic alterations with clinical importance in several NHLs.

### 2.2. Genetic Alterations with Prognostic Significance in Non-Hodgkin Lymphoma

Understanding the mutational status of lymphomas has not only improved diagnostics but has also led to a better risk stratification. Mutations in the *TP53* gene have been known to be associated with oncogenesis in a wide series of malignancies [29]. Its prognostic influence in lymphomas has also been proven in several entities [30]. In particular, patients with DLBCL [31,32], MCL [33] and CLL [34] harboring *TP53* mutations are known to have poorer prognoses. However, accessing *TP53* mutations is not established for all entities [30]. As an example, the International Prognostic Index (IPI), which is still the most important and widely used tool for risk stratification in DLBCL, does not include mutational status as a prognostic factor [35]. Moreover, newer scoring systems (R-IPI and NCCN-IPI) do not include mutational status either [36,37].

On the other hand, the CLL-IPI is a good example of the incorporation of mutational status together with clinical factors to discriminate between prognostic subgroups. The five factors of the CLL-IPI include the presence or absence of *TP53* mutations/del(17p), IGVH mutational status (mutated versus non mutated), serum β2-microglobulin concentration (≤3.5 mg/L vs. >3.5 mg/L), clinical stage (Binet A or Rai 0 vs. Binet B–C or Rai I–IV) and age (≤65 years vs. >65 years) [38]. Other known mutations known to impact prognosis are del(11q) */*ATM* mutations, trisomy 12, a complex karyotype and del(13q). In addition, NGS studies have helped to identify other mutations in CLL with prognostic importance, such as *BIRC3*, *NOTCH1* and *SF3B1* [39].

In multiple myeloma there are three main chromosomal aberrations, which are associated with worse outcomes and are classified as high-risk alterations. del(17p), t(4;14) and t(14;16) are included in the Revised International Staging System (R-ISS) [40]. Nevertheless, several other mutations have already been correlated with prognoses [41].

MCL has been known to have ambiguous clinical behavior. Research on the genetic level has helped to characterize the influence of mutational status on the development of a more severe disease course [39]. The MCL variant with a leukemic course often presents as a milder disease course. This variant shows a hypermutated IGHV, lacks a complex karyotype and does not necessarily require treatment at the time of diagnosis. In contrast, the more aggressive variant of MCL usually presents with a more complex karyotype, and may express SOX11 [40].

Identifying the genetic profile of aggressive lymphomas has also increased the understanding of prognoses and treatment response. Gene expression profiling of DLBCL using a specialized microarray identified two distinct molecular subtypes of lymphomas based on gene expression patterns indicative of different stages of B cell differentiation and so-called cell of origin (COO): activated B-cell-like (ABC) DLBCL, which expresses genes that are normally induced through the in vitro activation of peripheral blood B cells, and germinal center B-cell-like (GCB) DLBCL, in which genes share characteristics of germinal center B cells [42]. The current gold standard method for gene expression profiling (GEP) uses microarrays on RNA derived from frozen tissue (FT) [42]. Hence, this requirement limits its use in daily clinical practice. In addition, immunohistochemistry (IHC)-based algorithms have been proposed; however, the results are often inconsistent and present gaps in comparison to the standard procedure [43,44]. More recently, new strategies using formalin-fixed paraffin-embedded (FFPE) tissue biopsies have shown promising results [44,45].

Retrospective studies have shown that COO classification may impact prognosis. Several reports have demonstrated a significantly worse prognosis for patients with ABC DLBCL in comparison to GCB DLBCL [42,46,47]. However, these data were not confirmed in an analysis of two large prospective German trials [48], still leaving open questions and room for discussion. Some authors question the prognostic value of COO in comparison to other genetic profiling methods, especially the expression of BCL2 and MYC proteins, since these also correlate with COO [49]. Irrespective of the prognostic value of the COO classification, ABC and GBC lymphomas present with different phenotypes, and are recognized in the WHO classification as two separate entities [1]. On the other hand, the expression of MYC alone or BCL2 has been repeatedly correlated with worse prognoses in DLBCL [49,50,51].

Differently from tumors with an immunohistochemical expression of *MYC* and *BCL2* (double expressors), aggressive B-NHLs harboring *MYC* and *BCL2* or *MYC* and *BCL6* rearrangements—when identified with FISH—present an even more aggressive behavior, and are also referred to as double-hit lymphoma [52]. The revised WHO classification separated this entity from others, classifying it as a high-grade lymphoma. The presence of rearrangements in all three of the genes (MYC, BCL2 and BCL6) characterizes a further subgroup of high-grade lymphomas called triple-hit lymphoma [1].

However, in the past few years, three working groups using multiplatform genetic and expression classifiers have introduced molecular classifications for DLBCL with distinct prognoses. Schmitz et al. performed a multiplatform analysis of biopsy samples from 574 DLBCL patients in order to identify genetic subtypes of DLBCL based on shared genomic abnormalities [53]. They were able to identify four genetic subtypes, termed MCD (co-occurrence of *MYD88* p.L265P and *CD79B* mutations), BN2 (*BCL6* fusions and *NOTCH2* mutations), N1 (*NOTCH1* mutations) and EZB (*EZH2* mutations and *BCL2* translocations). The MCD and N1 subtypes showed significantly worse outcomes. The MCD and N1 subtypes were dominated by ABC cases, EZB corresponded mainly to GCB cases and BN2 had a mixed character [53].

In a similar manner Chapuy et al. performed a comprehensive genetic analysis of 304 primary DLBCLs in order to further stratify the disease according to genetic identity and its correlation with outcomes [54]. They were able to identify further DLBCL subsets: low-risk ABC DLBCLs of extrafollicular/marginal zone origin; two distinct subsets of GCB DLBCLs with different outcomes and targetable alterations; and an ABC/GCB-independent group with biallelic inactivation of *TP53*, CDKN2A loss and associated genomic instability. These subsets were correlated with prognoses independently from IPI [54].

Moreover, Wright et al. developed a probabilistic algorithm (LymphGen) to classify DLBCL into molecular subtypes based on the classifier of Schmitz et al. They identified two additional clusters: cases with *TP53* inactivation and those with recurrent mutations in *TET2*, *P2RY8* and *SGK1* genes, which were assigned to the A53 and ST2 clusters, respectively, leading to a total of six genetic subtypes [55].

### 2.3. Genetic Alterations with Therapeutic Significance

In addition to the diagnostic and prognostic value of the genetic milieu of lymphomas, the identification of specific molecular characteristics may also help guide therapy.

The mutational status in patients with CLL has provided important information regarding the influence of certain mutations on responses to therapy [56]. Data based on phase III clinical trials showed that patients harboring del(17p13) * do have a worse response to chemotherapy [57]. The difference in response was, however, no longer observed in patients who received non-chemotherapy-based regimens, such as the combination of the BCL2 inhibitor venetoclax and Bruton’s tyrosine kinase (BTK) inhibitors, such as ibrutinib [58,59]. Therefore, current guidelines recommend testing for *TP53* mutations for all CLL patients who are in need of therapy. As mentioned above, other mutations are known to have a worse impact on the prognoses of patients with CLL. In addition to testing for *TP53* mutations, testing for the presence of an unmutated IGVH or a complex karyotype is also recommended in many guidelines, since these patients, similarly to those harboring *TP53* mutations, seem to have a poorer response to conventional chemotherapy, and should be considered to receive newer treatment regimens based on BTK or BCL2 inhibition [60,61,62].

As mentioned above, the *MYD88* p.L265P mutation is of special interest in diagnosing lymphoplasmacytic lymphoma (LPL). This mutation influences cell survival through BTK-induced activation of κB [25]. This comprehension lead to the use of the BTK inhibitor ibrutinib for the treatment of LPL, which showed positive results [63]. In addition, WHIM (warts, hypogammaglobulinemia, infections and myelokathexis)-like mutations in *CXCR4* have been shown in approximately 30% of LPL patients, and may influence therapy [64]. One open-label multicenter study evaluated the response to therapy with ibrutinib in LPL, considering the mutational status of *MYD88* p.L265P and *CXCR4*. The authors found higher response rates among patients with *MYD88* p.L265P and without *CXCR4* (100% overall response rate and 91.2% major response rate), followed by patients with both mutations (85.7% and 61.9%) and patients without any mutation (71.4% and 28.6%) [63]. Moreover, the presence of *MYD88* in DLBCL may also predict responses to ibrutinib [65]. Additionally, the BN2 and MCD genetic subtypes seem to rely on chronically active BCR signaling, and may thus also be vulnerable to BTK inhibition [54].

Even though therapy with the R-CHOP (rituximab, cyclophosphamide, doxorubicin, vincristine and prednisone) regime is the worldwide standard for patients with DLBCL [66], it is known that patients with high-grade lymphoma, whether double- or triple-hit, present with poorer survival curves when treated with this well-established immunochemotherapy regimen [67,68]. Considering this, some authors suggest the use of different strategies in treating these patients [69]. Even though the evidence is still scarce and lacks randomized phase III trials, there are some data that suggest that more intensive regimens such as R-EPOCH (rituximab, etoposide, prednisone, vincristine, cyclophosphamide and doxorubicin) may provide better results for this distinct group of patients [70,71].

Within T cell lymphomas, the standard of care for patients with nodal peripheral T cell lymphomas (PTCL) consists of induction therapy, mostly with CHO(E)P (etoposide, prednisone, vincristine, cyclophosphamide and doxorubicin) or A-CHP (brentuximab vedotin, prednisone, cyclophosphamide and doxorubicin) and subsequent consolidation with high-dose chemotherapy and autologous stem cell transplantation (ASCT) [72]. Anaplastic large-cell lymphoma (ALCL) is a subtype of PTCL that may present with the expression of anaplastic lymphoma kinase (ALK). ALK-positive and -negative ALCL shows distinct clinico-pathologic characteristics [1,73]. Current treatment guidelines do not recommend consolidation with ASCT in patients with ALK + ALCLs and low–intermediate risk (IPI I-II) [72].

In addition to predicting treatment responses, advances in molecular techniques have been of help in understanding resistance mechanisms, especially those related to targeted therapies, in order to offer effective alternative treatment [74]. Ibrutinib inhibits BTK through irreversibly binding to the C481 residue [75]. Resistance to ibrutinib has been identified in the context of NHL, especially CLL and MCL. One study using whole-exome sequencing of peripheral blood samples of patients resistant to ibrutinib identified one cysteine-to-serine mutation in the C481S protein, preventing ibrutinib from binding to BTK [76]. In addition, mutations affecting the BCR-signaling-related molecule PLCγ2 (R665W, L845F and S707Y) were also identified by the authors, which potentially correspond to gain-of-function mutations that allow B-cell-receptor-mediated activation independently of BTK [76]. Another study identified clonal expansion in three of five patients, in which del(8p) was present, and may collaborate as a resistance mechanism through reduced or absent TRAIL-induced apoptosis [77]. Similarly, patients with CLL or MCL undergoing treatment with venetoclax may also have resistance mechanisms. Amongst the identified mechanisms, mutations in *BCL2* may play an important role. One of the identified mutations (p.G101V) affects the BCL2 structure, reducing its affinity to venetoclax. Other mechanisms, such as the overexpression of the MCL1 protein and complex transcriptional reprogramming with alterations of cell energy/metabolic pathways, have also been identified [74,78,79].

## 3. Advances in Molecular Methods and New Perspectives in Clinical Practice

With advances in the understanding of the genetic environment in lymphomas, as well as the rapidly expanding use of such data in daily clinical practice, there is a growing need for new technologies that are able to deliver faster and broader analyses.

Traditional methods are useful tools, and their use is consolidated for the analysis of molecular alterations in lymphomas. As mentioned above, both larger alterations—such as aneuploidies and translocations—and smaller alterations—such as point mutations and small insertions or deletions—play a role in lymphomagenesis and are frequently interrogated to provide additional information for diagnostic, prognostic and therapeutic matters.

Due to this broad spectrum of genetic alterations, many methods have been used in order to identify these changes. Larger alterations are frequently identified through karyotype analysis, fluorescence in situ hybridization (FISH) or polymerase chain reaction (PCR) methods [13], whereas smaller alterations have been identified through Sanger sequencing, fragment analysis, PCR with allele-specific oligonucleotides and melting curve analysis [80]. Due to the limited number of analyzed genes, these methods are known as low throughput, and even though they are well-established, they are still very work-intensive and cannot provide analyses of multiple genes simultaneously [81].

### 3.1. Comparative Genomic Hybridization

A technique that has been widely used in order to provide genome-wide screening for copy number variations is comparative genomic hybridization (CGH) [82]. As with other cytogenetic methods, it uses metaphase chromosomes in order to compare the DNA of a patient/individual with a normal control. Both samples are labeled with fluorescent nucleotides of different colors and pooled with a 1:1 ratio [83]. Differences in colors are interpreted to provide copy number variations. Similar to other cytogenetic methods, it has limitations: it is time-demanding and its interpretation is limited to bases measuring 5–10 Mb for most clinical applications [82]. However, the emergence of microarray methods, which use probes of multiple oligonucleotides with different sizes that are manufactured to detect areas of interest, has provided the opportunity to improve resolutions and outputs [82,84]. The combination of techniques is called array CGH, and has been used in lymphoma research, e.g., to detect copy number variations related to chemotherapy responsiveness [85].

### 3.2. Single-Nucleotide Polymorphisms, Sanger Sequencing and Next-Generation Sequencing

Array technology has also enabled the analysis of single-nucleotide polymorphisms (SNPs). SNP arrays can be used to identify, with a high resolution, copy number variations (CNVs) and acquired copy neutral loss of heterogyzosity [86]. SNP arrays have been used in several hematological malignancies and have helped detect SNPs involved in lymphomagenesis [87], prognoses [88] and, moreover, has broadened the understanding of the mutational status of other lymphoma entities [86,89].

Sanger sequencing is a traditional and widely used technique for DNA sequencing that is based on the selective incorporation of radioactively or fluorescently labeled chain-terminating dideoxynucleotides by DNA polymerase during in vitro DNA replication. The resulting DNA fragments are heat-denatured and separated by size using gel electrophoresis [90]. The Sanger method is restricted to the discovery of substitutions as well as small insertions and deletions. Next-generation sequencing (NGS) also uses the addition of fluorescent nucleotides; however, it is able to perform parallelly with millions of fragments per run. This enables high-throughput sequencing, which means that a whole genome may be sequenced within a day [91].

As mentioned previously, NGS has enabled the discovery of many new genetic signatures in many oncological diseases, including lymphomas [23]. In addition, it allows for the evaluation of IgH rearrangements and other patient-specific mutations, which can be used as markers for accessing minimal residual disease (MRD), which is already established in leukemic diseases such as acute lymphatic leukemia (ALL) and acute myeloid leukemia (AML) [92,93], but has had growing importance in CLL and other hematological diseases such as MM [91].

NGS has also allowed for the emergence of other investigational methods which immensely profit from high-throughput sequencing, such as liquid biopsy [94].

### 3.3. Liquid Biopsy

Liquid biopsy is a noninvasive procedure that allows for the detection of evidence of tumors in the peripheral blood [94]. In recent years, much scientific effort has been invested in the development of liquid biopsy, since it may overcome some of the difficulties related with the obtention of tissue probes for pathological analysis, such as difficult anatomical access, patients in critical condition, an unjustifiable risk of surgery, limited sensitivity of imaging technics for follow-up, amongst many others [95,96]. There are several methods of detecting tumor traces in the blood. Evidence of a malignancy may be present through the direct identification of circulating tumor cells, or indirectly through the investigation of genetic material released from tumor cells: DNA, microRNAs and tumor-derived exosomes [96]. DNA can be detected in the blood of virtually all individuals, and it is frequently named cell-free DNA (cfDNA). The amount of detected DNA varies in the population; it is frequently higher in the presence of several pathologies, such as infections, but particularly so with cancer [81,97]. Hence, one portion of this DNA originates from healthy cells. However, in patients with malignancies another share of this material is shed from tumor cells during apoptosis or necrosis [80,97]. This portion of DNA that originates from malignant cells is known as circulating tumor DNA (ctDNA). This ctDNA can be sequenced by using targeted approaches, where recurrent changes may be analyzed, or through broader strategies, such as whole-genome or -exome approaches [80,81]. Nevertheless, the sensitivity of the capture and analysis of ctDNA is limited by the proportion of tumor genetic material in comparison to DNA originating from healthy cells [98]. In NHLs ctDNA has been detected in several entities, including BL, DLBCL, MCL, FL and ALCL [99,100,101]. Since whole-genome approaches are currently not feasible in daily clinical practice, the development of targeted strategies, in which a group of particular known genetic alterations can be tested in order to access ctDNA, is advancing [81]. Low-throughput methods may be used in order to recognize specific ctDNA; however, there is a need for patient-specific primers and the number of studied genes is limited, which reduces the range of clinical use for these methods [13]. By using high-throughput methods, the need for patient-specific primers is eliminated. In addition, NGS approaches are capable of investigating not only markers with potential use for evaluating MRD, but they also have the ability to interrogate potential resistance mechanisms and therapeutic targets in a very individualized manner [102]. Two high-throughput methods have been tested with promising results as tools in the evaluation of ctDNA in oncological diseases: immunoglobulin high-throughput sequencing (Ig-HTS) and CAncer Personalized Profiling by deep Sequencing (CAPP-Seq) [103,104]. Furthermore, due to the role of the Epstein–Barr virus (EBV) in lymphomagenesis, EBV viral cell-free DNA has been investigated in evaluating MRD. The Ig-HTS technique is particularly interesting for NHL, since all B cell NHLs present a unique DNA clonotype that originates through the junction of immunoglobulin variable, diversity and joining genes [105]. This approach has been shown to be capable of detecting MRD in samples of patients with DLBCL [105,106], and was able to correlate with prognoses in patients with FL [107]. Ig-HTS assays may similarly be used for accessing MRD in liquid biopsy using DNA isolated from circulating tumor cells (CTCs) or bone marrow samples. In CLL IgHTS may be used to measure MRD using PBMCs [108]. Other leukemic diseases, such as MCL, may also be evaluated with IgHTS from CTCs. Similarly, MRD might be measured using DNA isolated from bone marrow samples of patients with MM [109]. Similar approaches may be used to identify and follow changes in TCR in diseases arising from T lymphocytes [104]. Table 2 summarizes non-invasive methods that are being investigated as noninvasive techniques in NHLs.

### 3.4. CAPP Sequencing

CAPP-Seq-based approaches, on the other hand, aim to detect several known mutations in a certain tumor entity with a broader clinical implication, since its use surpasses the identification of MRD [110]. In DLBCL, a research group investigated the use of a CAPP-Seq strategy, which included a DLBCL-focused sequencing panel that targeted recurrent single-nucleotide variations (SNVs), insertions/deletions and breakpoints, involving genes participating in canonical fusions (BCL2, BCL6, MYC and IGH), as well as IgVH and IgJH in several clinical landmarks [98]. This approach achieved a higher sensitivity compared to two studies based on IgHTS methods in a historical comparison [98]. In addition, CAPP-Seq was also useful for recognizing somatic alterations which emerged under treatment, including resistance mutations to ibrutinib, for predicting prognoses, for the early detection of relapse as a MRD tool, for characterizing tumors according to COO and finally for predicting transformations in FL [98]. In addition, a CAPP-Seq strategy was used in order to characterize tumors according to the four prominent genetic subtypes in DLBCL (according to Schmitz et al. 2018 [53]). The authors were able to describe two new classifiers (one COO classifier and a comprehensive genetic classifier including EZB, BN2, MCD, N1 and other, as defined by Schmitz et al.) [53] applicable to non-invasive plasma genotyping, which were tested in two independent data sets and able to predict outcomes [111].

Other methods for detecting ctDNA have also been used in lymphomas. They include whole-genome sequencing [112], amplicon-based sequencing of single-nucleotide variations and CNVs [113] in addition to digital-PCR-based approaches [110].

### 3.5. Viral Cell-Free DNA

EBV is a human gamma-1 herpesvirus whose etiological association to both lymphoproliferative lesions and malignant B, T and NK cell lymphomas is well-established [114]. In HL, tumor-specific molecular patterns in circulating cfDNA are currently lacking, mainly due to the lacking molecular characterization of the disease [115]. Thus, circulating EBV DNA may serve as a surrogate, as circulating pre-treatment EBV DNA reflects EBV positivity in tumor tissue and predicts treatment failure [116]. Furthermore, the persistence of cell-free EBV DNA at day eight predicts decreased event-free survival [115]. The prognostic significance of persistently detectable cell-free EBV DNA after treatment has also been demonstrated in NK/T cell lymphoma treated with the SMILE (dexamethasone, methotrexate, ifosfamide, L-asparaginase and etoposide) regimen [117].

### 3.6. Exosomes

Considering the growing applicability of liquid biopsy methods, the identification of circulating tumor cells/tissue has become an attractive research field. However, already in the late 1960s advances in electron microscopy led to the discovery of extracellular vesicles, whose clinical importance is yet to be fully explored [118]. Extracellular vesicles comprise a complex system of cell communication that takes place through the transport of cellular material within a vesicular membrane [119]. This is observed in a diversity of healthy tissues and systems, and it is involved in the maintenance of homeostasis [120]. However, they also seem to be involved in pathological pathways, including malignancies [121]. Exosomes are a particular class of extracellular vesicle that originate from multi-vesicular bodies with a size between 30–100 nm. They carry a diversity of contents, including RNAs and DNA, which correlate to important features of the parent cell [119]. Technical advances have enabled the isolation of exosomes, which has led to the understanding of the many roles that these particles exert in the pathogenesis of malignancies, especially hematological malignancies [122]. Exosomes may interfere with anti-tumor immunity through interacting with immune cells, change the bone marrow environment due to the effects of its contents (especially microRNAs) in cells that are exposed to it and are also involved in drug resistance, as they can interfere with immunotherapies [118]. Of special importance are the possible implications of exosomes for hematological malignancies regarding liquid biopsy, including diagnostics and MRD, due to the plethora of information carried by them [123].

### 3.7. MicroRNA

Understanding and recognizing genetic alterations, whose transcription generates functional proteins that have a direct influence on oncogenesis, has been a focus of research for many years [124]. Many of the alterations discussed above, exemplary rearrangements involving *MYC*, or del17p culminating in *TP53* alterations, are distinct examples. However, even though non-coding genetic material was not classically recognized as a central part of transcription and translation processes according to the central dogma of molecular biology [125], the role of non-coding genetic material, especially RNAs, has gained growing attention [126]. MicroRNAs (miRNAs) are one of the many non-coding RNAs that play an important role in regulating gene expression. Up- or downregulation of miRNAs has been associated with lymphomagenesis [127]. MiRNAs bind to target mRNAs and induce gene silencing, a function that is involved in almost all cellular processes [128]. Hence, alterations in miRNAs may lead to the dysregulation of the expression of important proteins, with direct consequences for tumor growth and development [124]. This was first recognized in CLL, where 13q14 deletions, a frequent alteration in CLL [39], were found to be associated with a downregulation of miR15 and miR16 [129]. This downregulation is possibly correlated with the de-repression of oncogenes and upregulation of TP53 mRNA [130]. Nowadays, many other miRNAs have been correlated with a diversity of NHLs, showing prognostic and potential therapeutic importance [131]. For example, the overexpression of miR-155 is increased in FL, CLL and DLBCL, especially ABC DLBCL [127,132]. Other examples of miRNAs with a proven association with NHLs and lymphomagenesis are miR-17-9, miR-21, miR-150 and miR-34a [127]. The clinical applications of this emerging technique are promising, since miRNAs cannot only be diagnosed in biopsy material but also in serum, plasma and bone marrow, and probably have the possibility as a potential target for liquid biopsy analysis [126]. One group of researchers correlated serum levels of miR-224, miR-455-3p, miR-1236, miR-33a and miR-520d-3p with responses to classic chemotherapy [133]. More recently, one study analyzed the plasma repository samples of 16 blood donors taken months or years before the diagnosis of DLBCL [134]. They observed upregulated microRNAs at the time of diagnosis, namely miR-328, miR-21-5p, miR-326 and miR-199a-5p. Interestingly miR-326 and miR-199a-5p were also upregulated in the last sample before diagnosis, indicating that these miRNAs were already upregulated in the absence of symptoms or diagnosis [134]. Similarly, miR-375 was found to be most down-regulated in DLBCL patients. This microRNA was also downregulated in some, but not all, repository samples before diagnosis, demonstrating a potential role of miRNAs as a diagnostic tool. The clinical relevance of this and other techniques using miRNAs still need to be validated in a larger data setting; however, it definitely comprises an attractive target for diagnostic, prognostic and even therapeutic purposes [131].

## 4. Future Directions

With the above-mentioned upcoming molecular techniques for lymphomas, the question arises as to which technique is suitable for diagnosis, research and the detection of MRD. As lymphomas are heterogeneous diseases there is no general answer for this challenging question. Leukemic lymphomas, such as CLL, can be easily diagnosed using flow cytometry due to the abundant presence of CTC. This technique also allows for the detection of MRD, which has been well-established in clinical practice. Furthermore, CTCs allow for the detection of clonal evolution, which may be involved in therapy resistance and disease progress.

In other lymphomas, e.g., nodal lymphomas, different techniques are needed. Whereas ctDNA may overcome some of the challenges related to classic methods, such as the need for invasive procedures to obtain tissue samples, this will need to be shown in the future, as of now there are not enough data supporting its use for diagnostic purposes only. Moreover, in many cases, ctDNA might not be detectable in patients with early stage cancer, or even in patients in advanced stages. In addition, even with the detection of ctDNA, not all detected mutations have clinical relevance [135]. One particular challenge for this application in lymphomas might be their heterogeneous clinical presentation, which can in many cases mimic other malignancies, especially when extra nodal involvement is present. This would imply extensive genetic investigations, which could be too expensive and work-intensive; however, advances in sequencing techniques, such as NGS combined with extensive databases, may enable their use for diagnostics and screening in the future. Nevertheless, establishing diagnoses using tissue samples will most likely remain the gold standard for these entities (lymphomas) for the next decade. Of note, despite the increasing use of minimal invasive methods such as endoscopic biopsies and core needle biopsies, especially combined with other methods such as flow cytometry, the established excisional surgical lymph node biopsy still remains the methods of choice—when clinically applicable—since it allows for a complete evaluation of the lymph node architecture, reducing the probability of false negative results and enabling pathological diagnosis. One exception could be cases with high clinical suspicion and high pretest probability, narrowing down the number of investigated genes. Nevertheless, it is extremely important to differentiate between the several lymphoma entities, which would likely require a multigene assay with clinical validation. Considering the high variability in lymphomas, this still requires an enormous amount of genetic data of several lymphoma subtypes, whose characterization is still an ongoing process.

However, if a lymphoma diagnosis is established, liquid biopsy via ctDNA might be a suitable technique to capture the average of a patient’s tumor mass, including its clonal architecture. This is of special interest as a conventional re-biopsy might not reflect the temporal heterogeneity of the disease, including the emergence of treatment-resistant clones. In addition, monitoring the dynamics of ctDNA over time may also be an interesting tool. Studies of ctDNA in DLBCL using CAPP-Seq have demonstrated promising results in the early detection of relapse in patients that underwent treatment. Scherer et al. were able to detect relapse using ctDNA before clinical evidence of relapse, even with considerable small amounts of detectable ctDNA, which confers its capability for MRD detection [98]. This may be particularly relevant in daily clinical practice when combined with imaging methods, especially FGD-positron emission tomography. Whereas the evidence of miRNAs in lymphomas has been growing and arises as a possible addition in the field of non-invasive diagnostics, their clinical application still needs more comprehensive data and validation. In particular, their possible therapeutic implications makes them an interesting target for research purposes. Additionally, the same applies for exosomes.

## 5. Conclusions

Advances in molecular methods have not only allowed for the expansion of knowledge regarding lymphomagenesis, but also have increased the variety of tools that are used in daily clinical practice, including diagnosis, risk stratification, identification of therapeutic targets, treatment resistance and establishing MRD measurements in lymphomas. Nevertheless, even though significant progress has been made in recent years, many methods still need to be validated or are yet limited to experimental use. However, with cost reduction and increasing availability, newer methods are going to significantly impact clinical approaches on patients with lymphomas.

## Figures and Tables

**Table 1:** Genetic alterations with consolidated clinical implications in NHL.

Disease	Genetic Alteration	Clinical Significance
Burkitt lymphoma	*MYC* rearrangements:	Diagnosis
	t(8;14)(q24:q32),	
	t(2;8)(p12;q24) and	
	t(8;22)(q24;q11)	
Burkitt-like lymphoma with 11q aberrations	Aberrations in the 11q chromosome and no *MYC* rearrangements	Diagnosis
Follicular lymphoma	Rearrangement of BCL2 gene,	Diagnosis
	t(14;18)(q32; q21)	
Mantel cell lymphoma	Rearrangement of the cyclin D1 gene,	Diagnosis
	t(11;14)(q13;q32)	
	TP53, complex karyotype, IGHV status and SOX11	
		Differentiation between aggressive and non-aggressive subtypes, possibly impacting therapy decision
	C481S	Therapy (ibrutinib resistance)
Hairy cell leukemia	BRAF V600 E/K	Diagnosis
Hairy cell leukemia variant	MAPK1	Diagnosis
Lymphoplasmacytic lymphoma	MYD88 p.L26P	Diagnosis and therapy
	CXCR4	Therapy
Chronic lymphocytic leukemia	TP53/del(17p)	Prognosis and therapy
	IGVH mutations status	
	Del(11q)	
	Del(13q)	
	Trisomy 12	
	Complex karyotype	
	C481S	Therapy (Ibrutinib resistance)
Multiple myeloma	del(17p) t(4;14) and t(14;16)	Prognosis/risk stratification and choice of treatment
Diffuse large B cell lymphoma	MYC or BCL2	Prognosis
	TP53	Prognosis
High-grade lymphoma	MYC and BCL2, MYC and BCL6 or MYC, BCL2 and BCL6	Diagnosis and therapy
Anaplastic large-cell lymphoma	ALK	Therapy

**Table 2:** Non-invasive molecular methods for NHLs.

Method	Possible Clinical Implications	Disadvantages
Circulating-tumor-DNA-based CAPP sequencing; Ig-HTS; Whole-genome sequencing; Digital PCR; SNV and CNV.	Diagnosis, risk stratification, follow-up (MRD and response to treatment) and directing therapy	Not commercially available; Only suitable for MRD; Low cost and time effectiveness, not suitable for MRD; Low throughput, depends on hotspots; Lower sensitivity.
Circulating-tumor-cells-based	Diagnosis, risk stratification, follow-up (MRD and response to treatment) and directing therapy	Limited sensitivity for non-leukemic NHLs
Micro-RNA	Diagnosis, risk stratification, follow-up (MRD and response to treatment) and directing therapy, possibly as a therapeutic approach	Not yet clinically stablished
Exosomes	Diagnosis, follow-up, directing therapy and possibly as a therapeutic approach	Limitations of technique: difficulties in isolating exosomes. Not yet clinically established
Detection of viral cell-free DNA	Follow up (MRD and response to treatment)	Available for a limited number of NHLs

CAPP—CAncer Personalized Profiling; Ig-HTS—immunoglobulin high-throughput sequencing; PCR—polymerase chain reaction; SNV—single-nucleotide variation; CNV—copy number variation; MRD—minimal residual disease; and NHL—non-Hodgkin lymphoma.

## Data Availability

Not applicable.
